# Supplementary material for: Increased slow dynamics defines ligandability of BTB domains
Source: Nat Commun. 2022 Nov 16;13:6989. doi: 10.1038/s41467-022-34599-6 (PMC9668832; doi:10.1038/s41467-022-34599-6)
Supplement: Supplementary file 1 — Supplementary Info File #1 [file 41467_2022_34599_MOESM1_ESM.pdf]

# Supplementary Information

for

**Increased slow dynamics defines ligandability of BTB domains**

Vladlena Kharchenko[1,#], Brian M. Linhares[2,#,$], Megan Borregard[2], Iwona Czaban[1], Jolanta Grembecka[2], Mariusz Jaremko[1], Tomasz Cierpicki[2,*] and Łukasz Jaremko[1,*]

[1]Smart Health Initiative (SHI), Red Sea Research Center (RSRC), Bioscience Program, King Abdullah University of Science and Technology (KAUST), Biological and Environmental Science & Engineering (BESE), 23955-6900 Thuwal, Saudi Arabia

[2] Department of Pathology, University of Michigan, 1150 West Medical Center Dr, MSRB I, Room 4510D, Ann Arbor, MI 48108, USA.

[#] equal contributions

[$]current address: Siduma Therapeutics, Inc. 55 Church St., 8th Fl. New Haven, CT 06510

* corresponding authors' e-mails: lukasz.jaremko@kaust.edu.sa, tomaszc@umich.edu

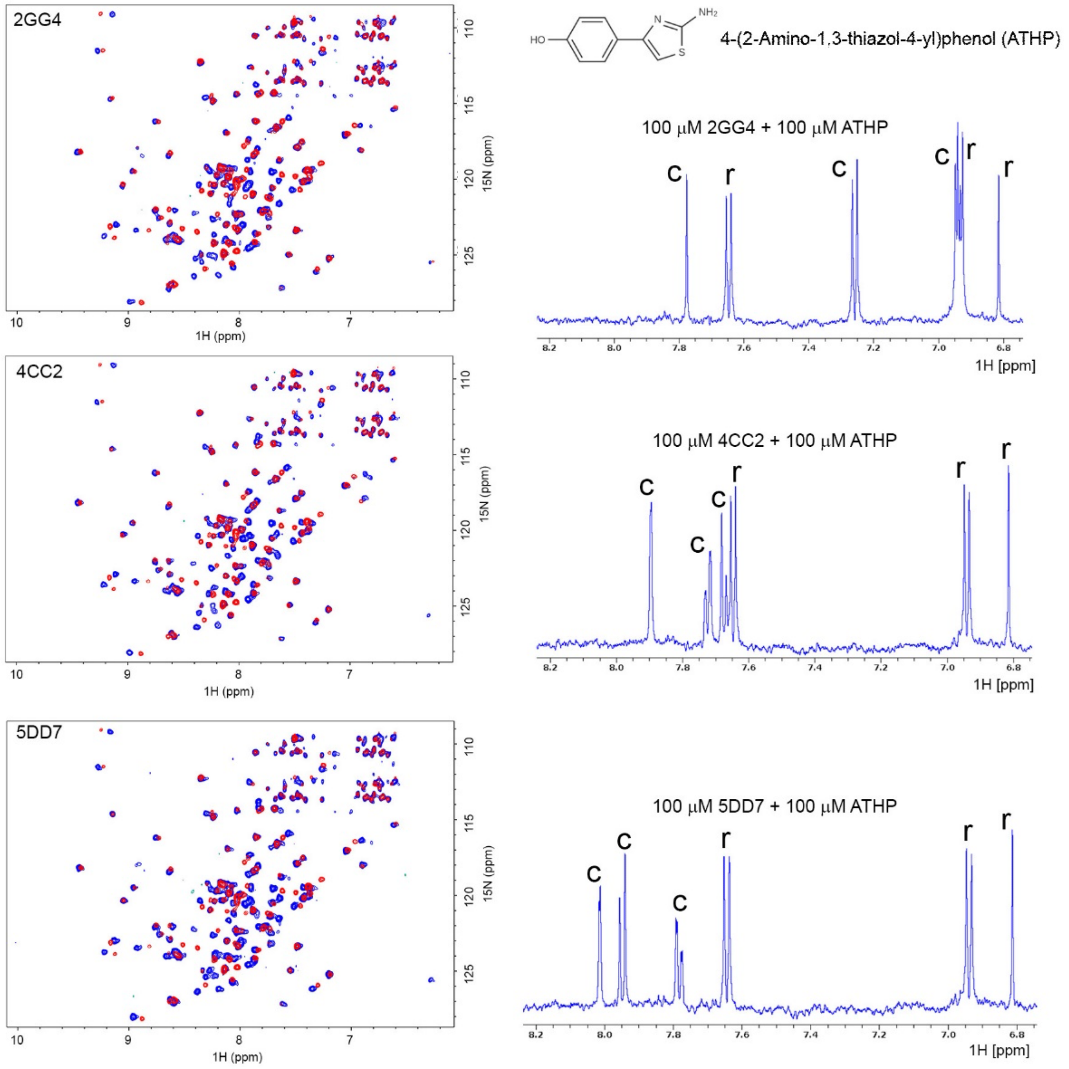


**Supplementary Figure 1. FBS-derived and NMR-identified highest scoring fragments |** The 2D [$^{1}$H-$^{15}$N] HSQC spectra of MIZ1$^{BTB}$ with screening mixtures containing: 2GG4, 4CC2 and 5DD7 hits, respectively. Spectra were collected at 150 µM uniformly labeled U-[$^{15}$N] MIZ1$^{BTB}$ in buffer comprised of 50 mM Tris (pH 7.5), 150 mM NaCl, 1 mM TCEP, 7.5% $D_2O$ (*v/v*), and 5% DMSO (*v/v*), in absence (blue) and presence of 1 mM compound (red). The 1D $^{1}$H NMR spectra represent the experiments used to calibrate concentrations of fragment compounds. The NMR spectra were recorded at a 600 MHz NMR instrument for samples containing 100 µM compounds mixed with 100 µM ATHP reference compound (ATHP structure is shown in the figure) in 50 mM Tris (pH 7.5), 150 mM NaCl, 1 mM TCEP, 7.5% $D_2O$ (*v/v*), and 5% DMSO (*v/v*) buffer at 30°C. Signals from tested compounds are labelled “c”, and the ATHP reference signals are labelled “r”.

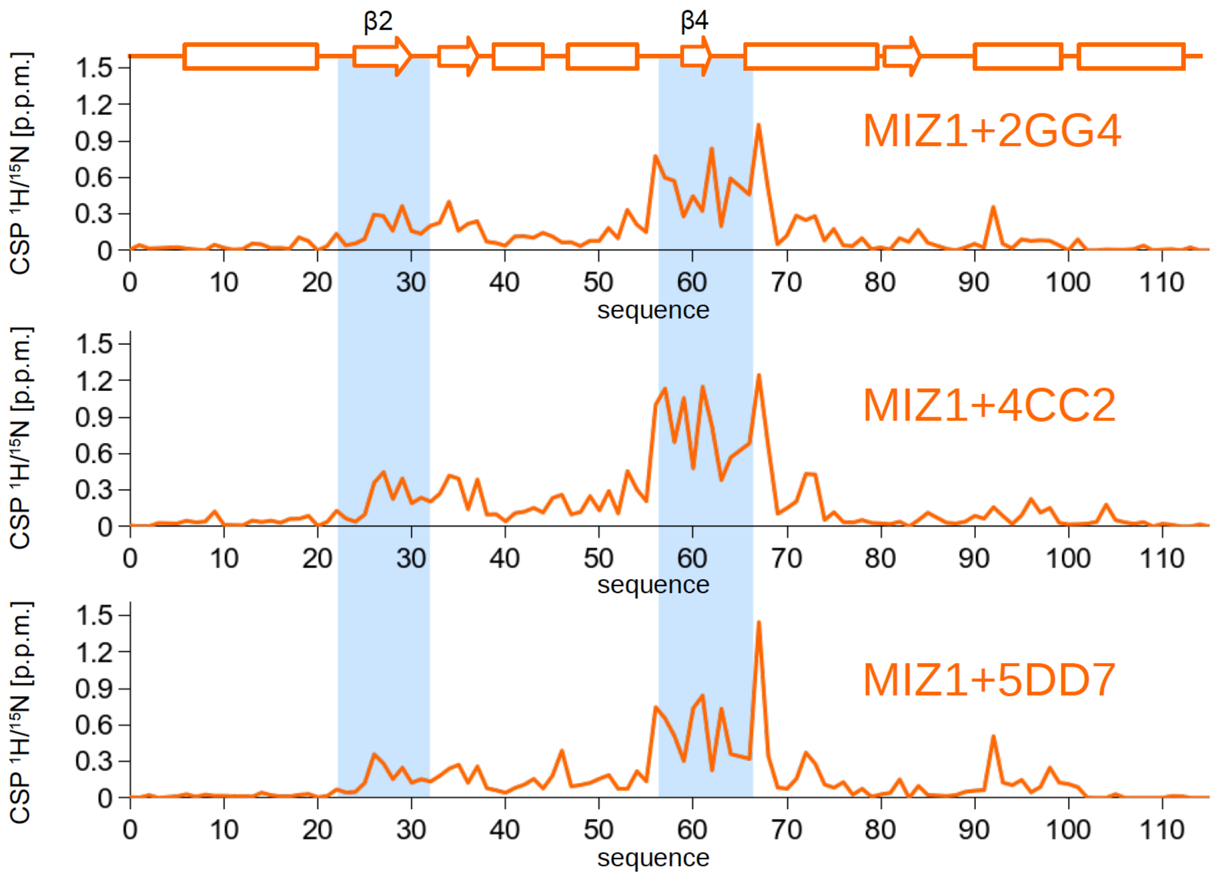


**Supplementary Figure 2. The three most potent fragment compounds induce large chemical shift perturbations upon binding to MIZ1$^{BTB}$ and share a common binding site. |** The $CSP_{N/H}$ plots of MIZ1$^{BTB}$ and three compounds 2GG4, 4CC2 and 5DD7. The concentration of U-[$^{15}$N] labelled MIZ1$^{BTB}$ was 250 μM and 2GG4 1000 μM, while 4CC2 and 5DD7 were 2000 μM. The reference MIZ1$^{BTB}$ sample was 250 μM, all contained 5% DMSO (*v/v*), pH 7.5, 150 mM NaCl, 1 mM TCEP. The $CSP_{N/H}$ were calculated for the last titration point using the equation: $CSP_{H/N,i} = [(\Delta\delta_{H,i})^2+0.1\cdot(\Delta\delta_{N,i})^2]^{0.5}$. The blue shading indicates the position of B2 and B4 motifs. Source data is provided as a Source Data file.

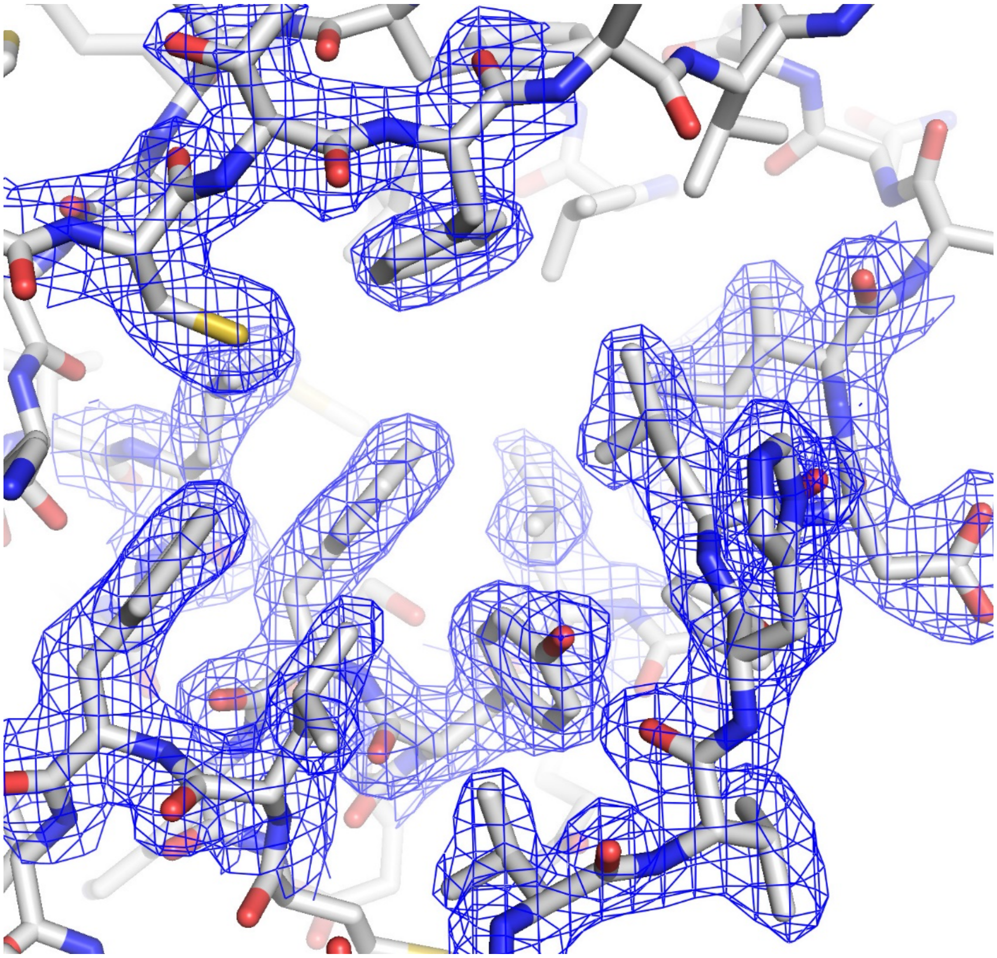

**Supplementary Figure 3. | Fragment of the crystal structure of MIZ1$^{BTB}$ domain.** Electron density is plotted as 2mFo-DFc map contoured to 1 $\sigma$, the backbone and side-chain non-hydrogen atoms are drawn as sticks; the PDB id: 7T58.

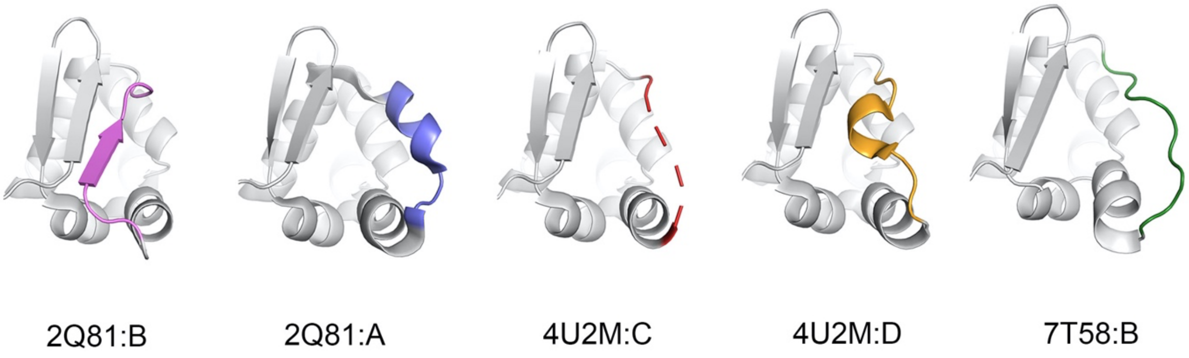


**Supplementary Figure 4. The B4 motif adopts a broad range of conformations in the crystal structures of MIZ1$^{BTB}$ |** Comparison of the crystal structures of selected MIZ1 BTB domain structures (labelled with PDB code and chain id) showing a variable B4 motif in different colors.

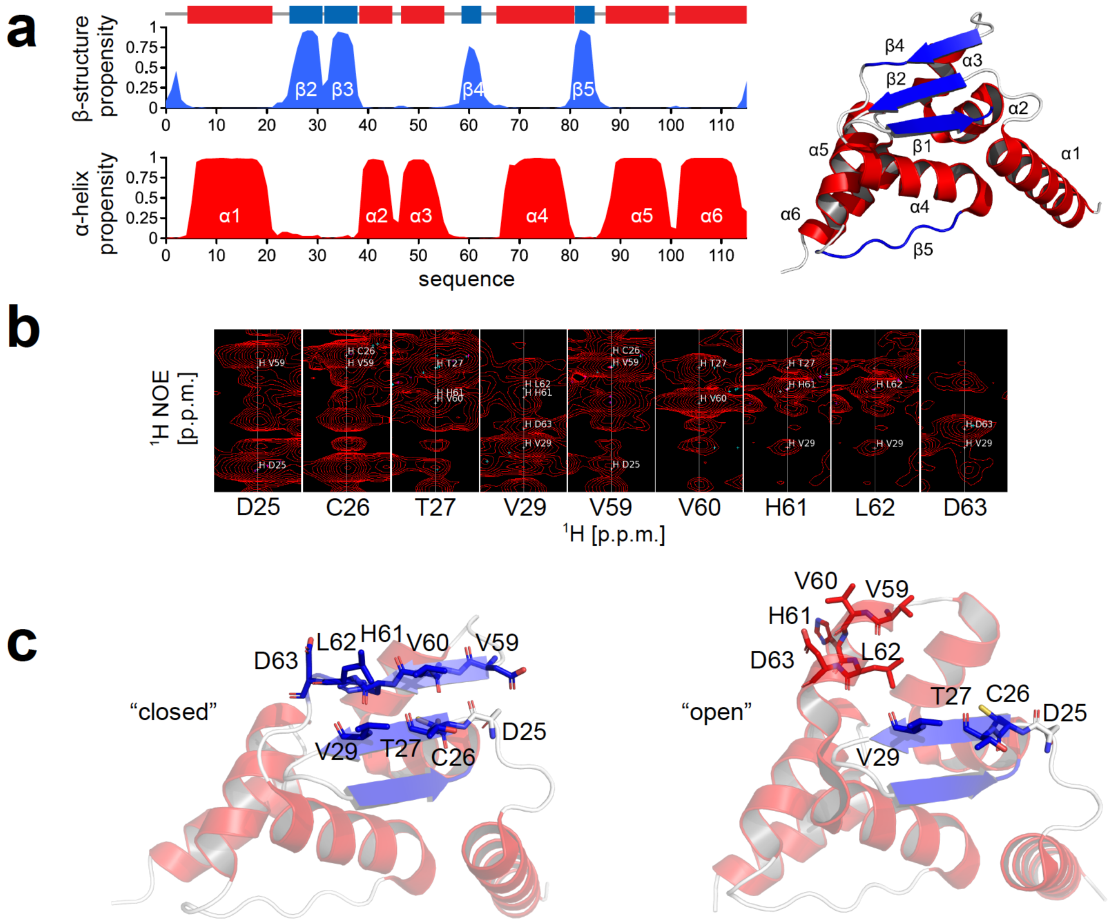


**Supplementary Figure 5. MIZ1$^{BTB}$ adapts the "closed" state in solution |** Combined analysis of chemical shifts and spatial $H^N$-$H^N$ connectivity derived in solution for uniformly double labeled U-[$^{13}C$,$^{15}N$] MIZ1$^{BTB}$ at pH 7.5, 150 mM NaCl, 1 mM TCEP. **a.** Propensities of the MIZ1$^{BTB}$ sequence to form secondary structure elements derived from backbone chemical shifts employing the Talos-N algorithm. Helices are marked red and β-strands in blue. The appropriate secondary structure elements are marked with Greek letters on the propensity profile and the adjacent 3D crystal structure of MIZ1$^{BTB}$ (PDB id: 2Q81:B). **b.** The $H^N$-$H^N$ NOE connectivity, *i.e.*, the through-space distances below 6 Å between the specified amide protons, identified for MIZ1$^{BTB}$ in 3D $^{15}N$-edited HMQC-NOESY (mixing time 100 ms). **c.** The B4 region adopts a "closed" conformation of the β4 strand (residues V59-D63) paired with β2 motif (residues D25-V29) (left; PDB id: 2Q81:B) in agreement with $H^N$-$H^N$ connectivity from 3D $^{15}N$-edited HMQC-NOESY spectrum, while the "open" conformation is not consistent with $H^N$-$H^N$ connectivity pattern (right; PDB id: 2Q81:A). Source data is provided as a Source Data file.

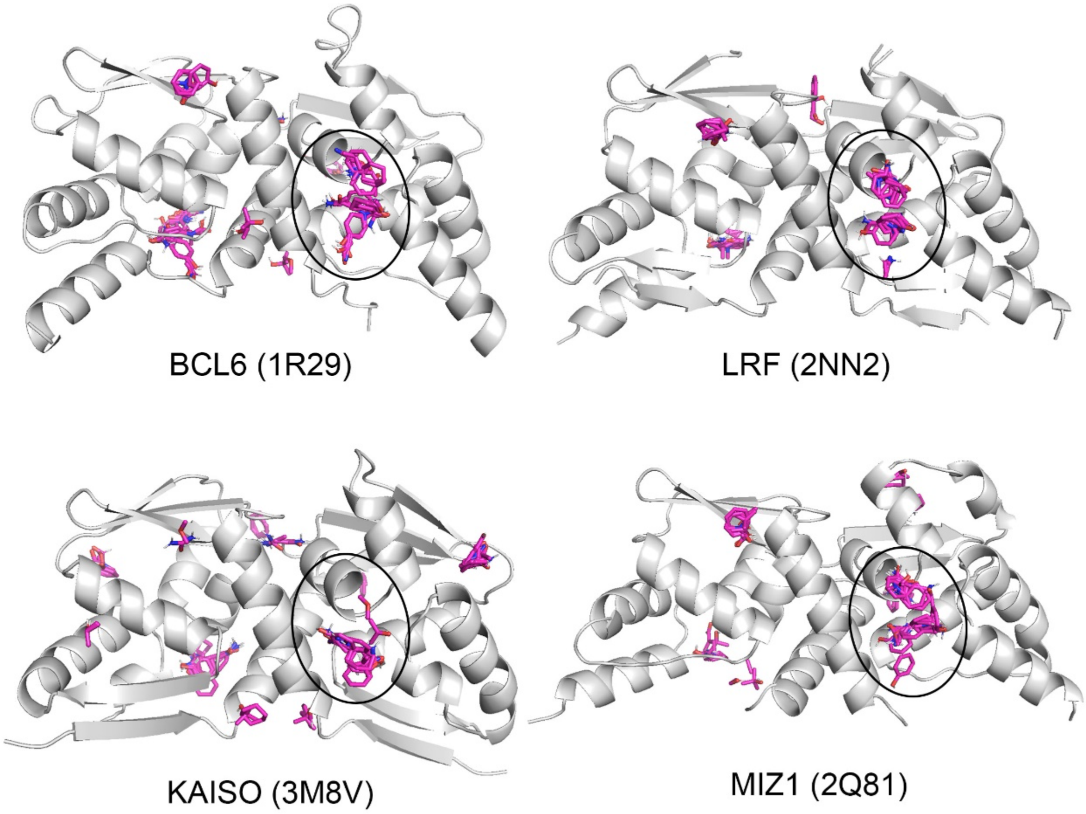


**Supplementary Figure 6. Mapping of ligand binding hot-spots on the structures of BTB domains calculated using FTMAP software |** The FTMAP (https://ftmap.bu.edu/) calculations were performed using default parameters and show the mapping of various probe compounds (magenta) using crystal structures of BTB domains with indicated PDB codes. The site encompassed by helices $\alpha2$, $\alpha3$, $\alpha6$ and $\alpha1$ from the second monomer represents a binding site of BCL6 BTB domain inhibitors is circled.

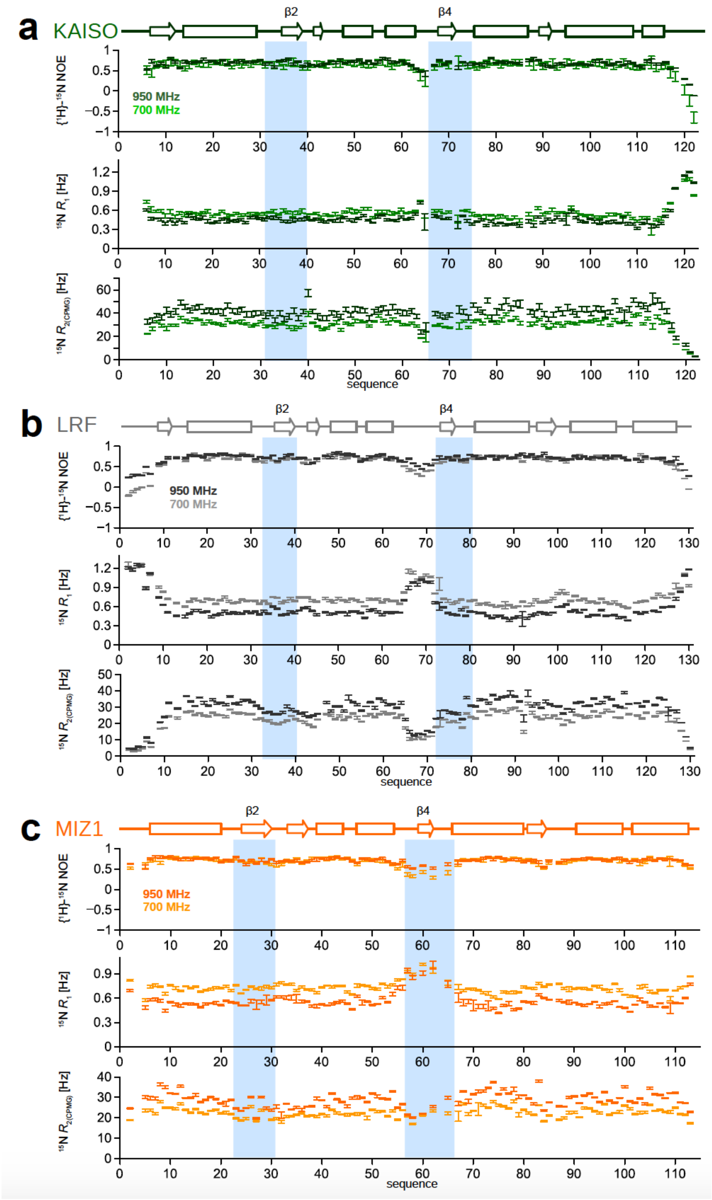


**Supplementary Figure 7. The BTB domains of KAISO, MIZ1 and LRF display distinct dynamic profiles** | The comprehensive set of $^{15}$N spin relaxation observables, at two magnetic field strengths, for KAISO$^{BTB}$ (a; green), LRF$^{BTB}$ (b; grey) and MIZ1$^{BTB}$ (c; orange). Shaded regions indicate the isostructural positions of β4 and β2 strands, secondary motifs are marked with rectangles (helices) and arrows (strands). The relaxation rates were fit from the intensities to exponential decay curves with two parameters in a non-linear least-square procedure, and standard errors were derived from variance-covariance matrix analysis. The {$^1$H}-$^{15}$N heteronuclear NOE values were obtained from the ratio of intensities and errors from the propagation of the signal-to-noise values of two signals corresponding to an individual residue. Source data is provided as a Source Data file.

| Protein | $MIZ1^{BTB}$ | $LRF^{BTB}$ | $KAISO^{BTB}$ | $MIZ1^{BTB}$:HUWE1 |
|---|---|---|---|---|
| MW [kDa][a] | 29.0 | 29.4 | 29.0 | 30.3 |
| $MW_{SEC\text{-}MALS}$ [kDa][b] | 24.8 (0.3) | 26.5 (0.2) | 28.2 (0.2) | N.D. |
| $\tau_R$ [ns][c] | 16.09 (0.09) | 16.75 (0.09) | 22.45 (0.45) | 24.12 (0.47) |
| $D_1$ [$10^6$ $s^{-1}$] | 10.17 (0.10) | 7.86 (0.06) | 7.40 (0.21) | 7.09 (0.22) |
| $D_2$ [$10^6$ $s^{-1}$] | 7.89 (0.09) | 10.20 (0.11) | 5.45 (0.19) | 5.14(0.17) |
| $D_3$ [$10^6$ $s^{-1}$] | 13.02 (0.10) | 11.79 (0.11) | 9.42 (0.35) | 8.51 (0.23) |
| $A$[d] | 1.44 (0.01) | 1.31 (0.01) | 1.46 (0.04) | 1.39 (0.05) |

[a] as calculated from primary sequence by ProtParam tool (https://web.expasy.org/protparam/);
[b] average MW as determined from triplicate measurements (mean ± S.D);
[c] total correlation time, $\tau_R = 0.5/(D_1+D_2+D_3)$, (errors are propagated from the diffusion constants uncertainties);
[d] anisotropy of the diffusion tensor, $A = 2D_3/(D_1+D_2)$, (errors are propagated from the diffusion constants uncertainties).

**SEC-MALS profiles**

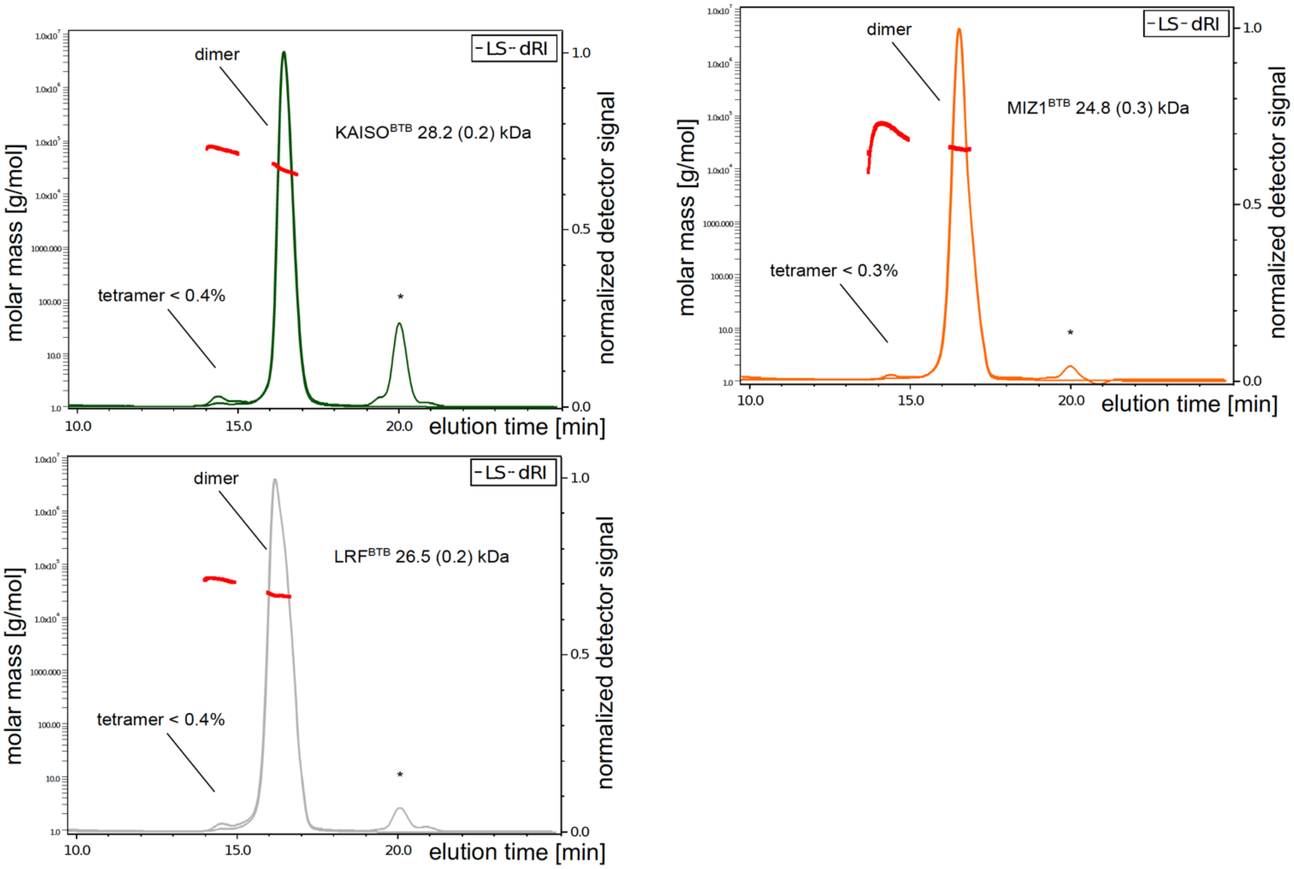


**Supplementary Figure 8. The BTB domains from MIZ1, LRF and KAISO are dimeric in solution. |** Molecular weights and the total correlation times of BTB domains in solution (table) as determined by $^{15}N$ spin relaxation studies at two magnetic fields and the size exclusion chromatography with multi-angle static light scattering (SEC-MALS). The SEC-MALS profiles for $KAISO^{BTB}$, $LRF^{BTB}$ and $MIZ1^{BTB}$ demonstrate a single (over 99.5%) signal representing the homodimer. The dRI stands for refraction index, while LS comes from a light scattering signal. The (*) mark indicates the small molecules, e.g., imidazole. Source data is provided as a Source Data file.

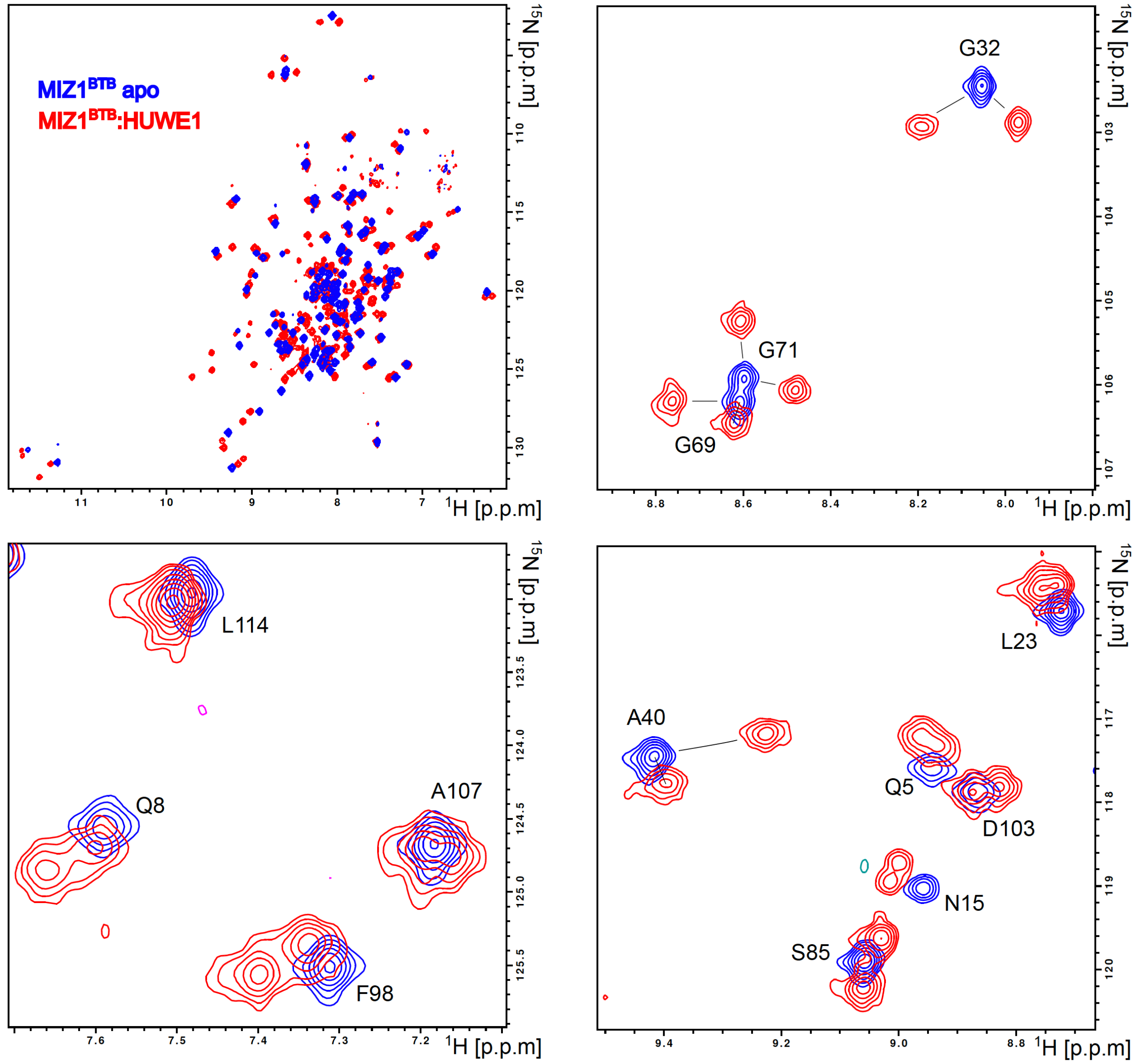


**Supplementary Figure 9. Binding to HUWE1 breaks the symmetry of the MIZ1$^{BTB}$ dimer |** The 2D [$^{1}$H-$^{15}$N] TROSY spectrum of 100 μM uniformly [$^{13}$C,$^{15}$N] labelled MIZ1$^{BTB}$ apo (blue) and 100 μM MIZ1$^{BTB}$:HUWE1 complex (1:3 molar ratio) pH 7.5, 150 mM NaCl, 1 mM TCEP and 1% $D_2O$ (*v/v*). The expanded fragments of the spectra with the resonance assignments are presented in the remaining panels. The spectra were recorded at 800 MHz NMR instrument at 30°C.

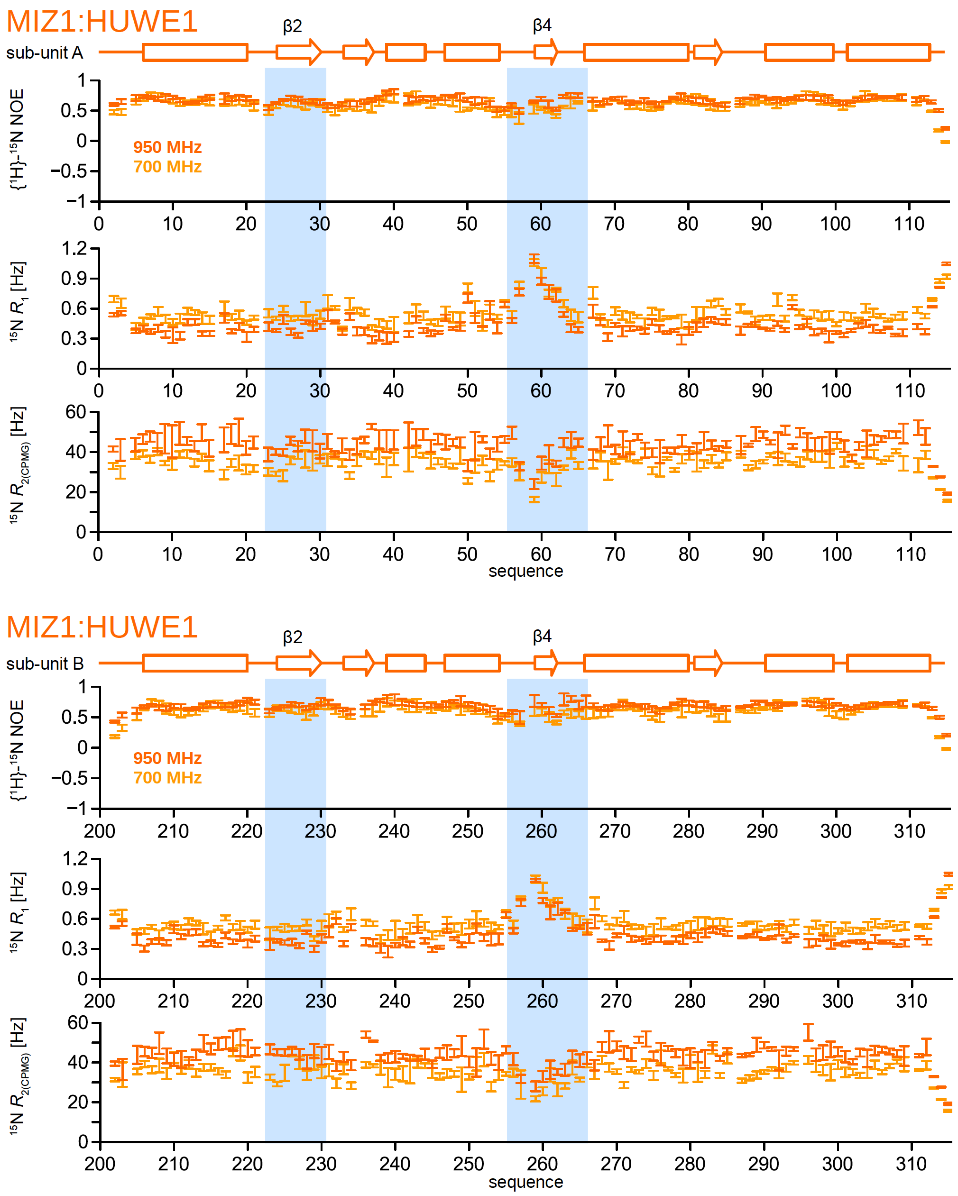


**Supplementary Figure 10. The binding of HUWE1 modifies the MIZ1$^{BTB}$ dynamic profile |** The $^{15}$N spin relaxation observables, at two magnetic field strengths, for MIZ1$^{BTB}$:HUWE1 complex. The shaded regions indicate the positions of β4 and β2 motifs, secondary motifs are marked with rectangles (helices) and arrows (strands). The relaxation rates were fit from the intensities to exponential decay curves with two parameters in a non-linear least-square procedure, and standard errors were derived from variance-covariance matrix analysis. The {$^1$H}-$^{15}$N heteronuclear NOE values were obtained from the ratio of intensities and errors from the propagation of the signal-to-noise values of two signals corresponding to an individual residue. Source data is provided as a Source Data file.

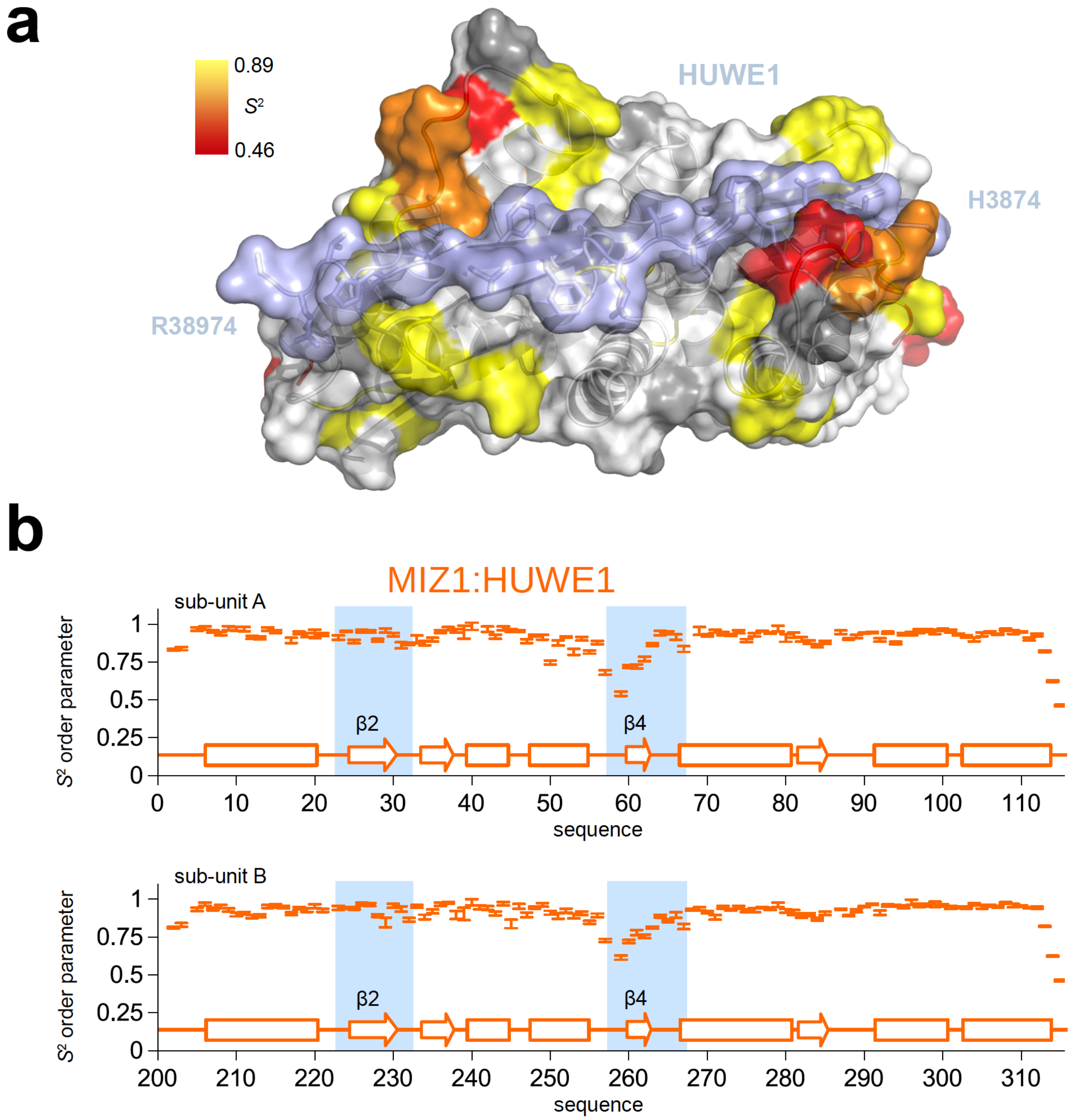


**Supplementary Figure 11. Analysis of the dynamics profile for the MIZ1$^{BTB}$:HUWE1 complex |** a) The $S^2$ order parameters mapped onto the structure of the MIZ1$^{BTB}$ domain complexed with HUWE1 peptide (sea-blue) scaled yellow to red to indicate increasing fast local dynamics (PDB: 7AZX). b) Order parameters $S^2$ reporting on the local fast dynamics plotted for the two chains in the asymmetric MIZ1$^{BTB}$ dimer. The secondary structure motifs are marked, and light-blue squares indicate B2 and B4 motifs. Values and corresponding errors are mean +/- SD obtained after Monte-Carlo procedure after 200 minimizations. Source data is provided as a Source Data file.

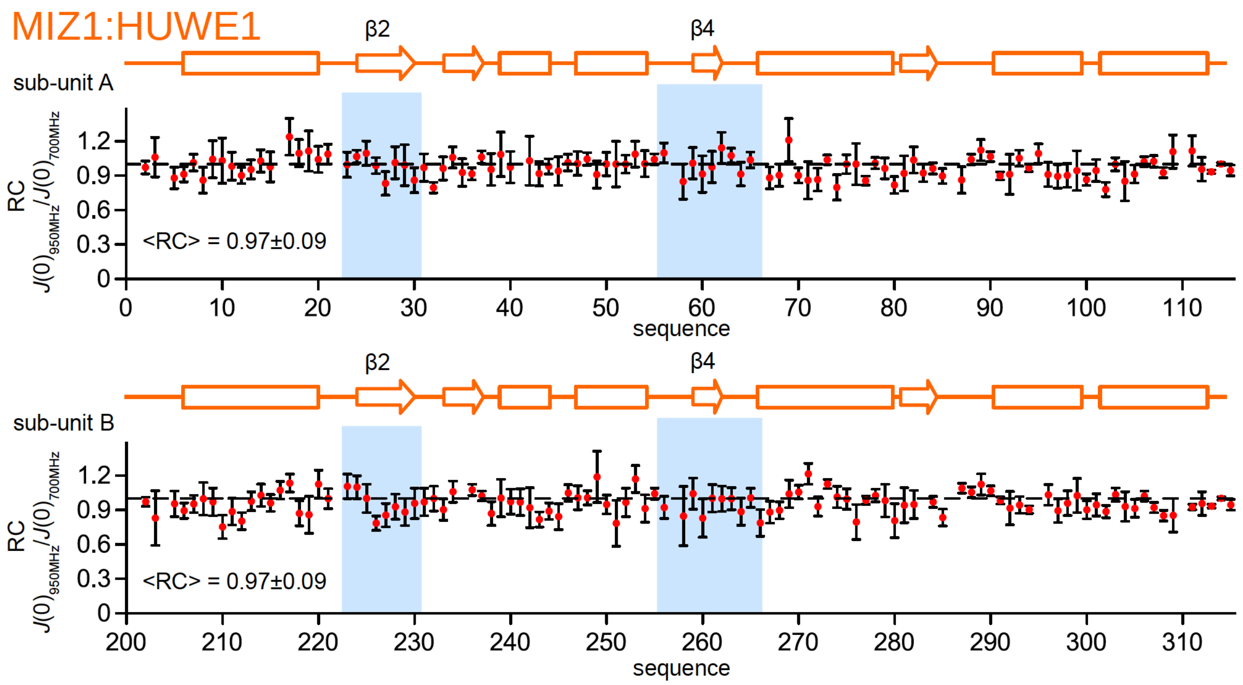


**Supplementary Figure 12. The RC$_i$ profile for the MIZ1$^{BTB}$-HUWE1 complex demonstrating a loss of µs-ms time scale dynamics within the B2 and B4 motifs in MIZ1$^{BTB}$ upon binding to HUWE1 |** The spectral density analysis plots derived from a comprehensive set of $^{15}$N spin relaxation observables determined at two magnetic field strengths for the uniformly labelled [$^{15}$N] MIZ1$^{BTB}$ bound to the unlabeled HUWE1 peptide. The RC$_i$ ratio errors are propagated from the spin relaxation observables' uncertainties. Shaded regions indicate the positions of β4 and β2 motifs. Source data is provided as a Source Data file.

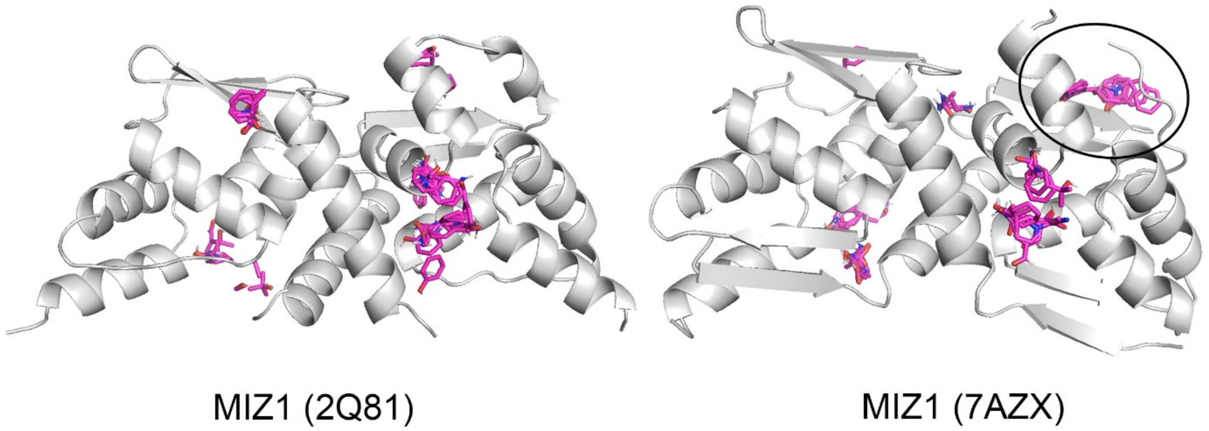


**Supplementary Figure 13. The FTMAP analysis of MIZ1 BTB domain in apo form (2Q81) and in complex with HUWE1 (7AZX).** FTMAP (https://ftmap.bu.edu/) calculations were performed using default parameters and show mapping of various probe compounds (magenta) including a new potential binding site (circled) identified in the structure of MIZ1 from the complex with HUWE1.

**Supplementary Table 1:** Summary of crystallographic statistics for MIZ1$^{BTB}$ domain.

| | Miz1 BTB domain (PDB 7T58) |
|---|---|
| **Data Collection** | |
| Space group | $C222_1$ |
| Cell dimensions | |
| *a, b, c* (Å) | 60.92, 115.90, 63.25 |
| α, β, γ (°) | 90.00, 90.00, 90.00 |
| Resolution (Å) | 41.30 – 2.05 (2.13 – 2.05) |
| $R_{merge}$ | 0.11 (0.44)* |
| CC1/2 | 0.988(0.914) |
| $\langle I \rangle / \langle \sigma(I) \rangle$ | 11.03 (2.66) |
| Completeness (%) | 91.4 (71.9) |
| Redundancy | 6.0 (5.2) |
| | |
| **Refinement** | |
| Resolution (Å) | 41.30 – 2.05 (2.13 – 2.05) |
| No. reflections | 79066 (5261) |
| $R_{work}$ / $R_{free}$ (%) | 18.2/23.2 |
| No. atoms | |
| Protein | 1734 |
| Ligand | 8 |
| Water | 216 |
| Mean *B*-factors (Å$^2$) | |
| Protein | 30.6 |
| Ligand | 35.6 |
| Water | 40.0 |
| r.m.s. deviations | |
| Bond lengths (Å) | 0.002 |
| Bond angles (°) | 0.43 |

*Values in parentheses correspond to highest resolution shells.
